# Soil Moisture and Excavation Behaviour in the Chaco Leaf-Cutting Ant (*Atta vollenweideri*): Digging Performance and Prevention of Water Inflow into the Nest

**DOI:** 10.1371/journal.pone.0095658

**Published:** 2014-04-18

**Authors:** Steffen Pielström, Flavio Roces

**Affiliations:** Behavioral Physiology and Sociobiology, Biocenter, University of Würzburg, Würzburg, Germany; Universidade de São Paulo, Faculdade de Filosofia Ciências e Letras de Ribeirão Preto, Brazil

## Abstract

The Chaco leaf-cutting ant *Atta vollenweideri* is native to the clay-heavy soils of the Gran Chaco region in South America. Because of seasonal floods, colonies are regularly exposed to varying moisture across the soil profile, a factor that not only strongly influences workers' digging performance during nest building, but also determines the suitability of the soil for the rearing of the colony's symbiotic fungus. In this study, we investigated the effects of varying soil moisture on behaviours associated with underground nest building in *A. vollenweideri*. This was done in a series of laboratory experiments using standardised, plastic clay-water mixtures with gravimetric water contents ranging from relatively brittle material to mixtures close to the liquid limit. Our experiments showed that preference and group-level digging rate increased with increasing water content, but then dropped considerably for extremely moist materials. The production of vibrational recruitment signals during digging showed, on the contrary, a slightly negative linear correlation with soil moisture. Workers formed and carried clay pellets at higher rates in moist clay, even at the highest water content tested. Hence, their weak preference and low group-level excavation rate observed for that mixture cannot be explained by any inability to work with the material. More likely, extremely high moistures may indicate locations unsuitable for nest building. To test this hypothesis, we simulated a situation in which workers excavated an upward tunnel below accumulated surface water. The ants stopped digging about 12 mm below the interface soil/water, a behaviour representing a possible adaptation to the threat of water inflow field colonies are exposed to while digging under seasonally flooded soils. Possible roles of soil water in the temporal and spatial pattern of nest growth are discussed.

## Introduction

Leaf-cutting ants of the genus *Atta*, a group native to the tropical and subtropical regions of the new world, live in large colonies of up to several million individuals that build remarkably large underground nests. Superficially, the nests can be recognised by large accumulations of excavated soil, often aggregated as a single nest mound. Numerous nest openings are used as exits/entrances by foragers that carry leaf material into the nest. The amount of soil deposited here can reach considerable dimensions. Autouri [1] estimated one nest mound of *Atta sexdens* (L.) to average about 40 metric tons of excavated material. In one *Atta laevigata* (Smith) nest, the excavated soil removed from the underground covered an area of 67.2 m^2^ [2]. Despite the impressive size that nest mounds can reach, the most important parts of the nest remain hidden underground. Here, leaf material is used as a substrate for a symbiotic fungus that is grown in subterranean fungus chambers [3]. Each nest has a large number of fungus chambers, up to eight thousand in a mature *A. laevigata* nest [2]. In most *Atta* species, additional chambers are built for refuse deposits [4]–[6]. Besides the nests of *Macrotermes* termites, those of the *Atta* leaf-cutting ants can be considered to be the largest insect-built structures in nature [7].

The Chaco leaf-cutting ant, *Atta vollenweideri* (Forel), is endemic to the Gran Chaco region and the areas around lower Río Paraná and lower Río Uruguay. Its nest architecture can be easily distinguished from that of other *Atta* species due to the ventilation turrets, erected on the central nest openings, that enhance wind-induced nest ventilation [8]–[10]. These are built with pellets excavated from clay-heavy alluvial alfisols generally encountered in the habitat of *A. vollenweideri* [11]. Studies in Paraguay demonstrated that *A. vollenweideri* does not occur in the red coloured soils in the eastern part of the country, where it is replaced by *A. laevigata* and *Atta capiguara* Gonçalves, but represents the only *Atta* species in the clayish soils that occur closer to the Río Paraguay and in the western part of the country [12],[13]. The only other *Atta* species that can be found in the Gran Chaco region is *Atta saltensis* Forel. It seems, however, that this species is restricted to patches of sandier soil and dry climate within the Chaco, e.g. the region of the Parque Nacional Copo (Santiago del Estero, Argentina), where no nests of *A. vollenweideri* occur (Roces, pers. observations).

The Gran Chaco region is generally characterised by high annual variability in moisture conditions. While the winters are mostly dry in the wet Chaco of the Formosa province in Argentina – total annual precipitation may reach up to 2000 mm, with an average annual minimum of ca. 40 mm in July – heavy rainfalls occur periodically in the spring and summer seasons. From October to April, precipitation is at least three to five times higher than the annual minimum [14].

**Figure 1:**
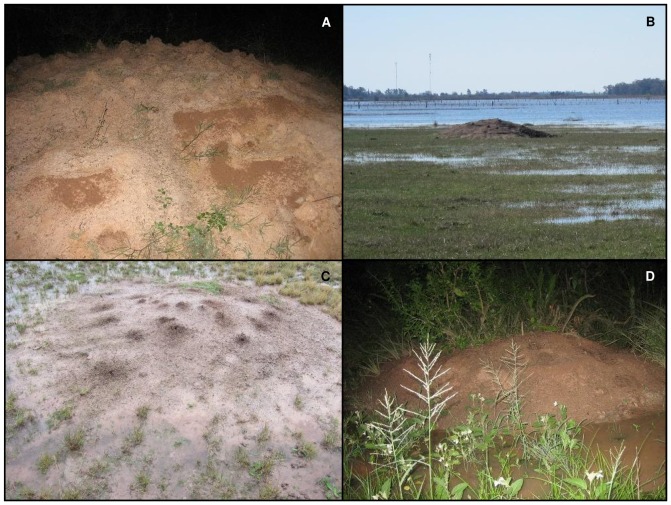
Water around and inside *A. vollenweideri* nests. (A) A nest mound at the Reserva El Bagual, Formosa, Argentina. Visible as a darker colour are the accumulations of freshly deposited, moist soil pellets carried up from the underground by workers during the night. The moisture of the freshly deposited pellets is often higher than that of the surrounding soil at the nest surface, giving them a darker colour. (B) *A. vollenweideri* nest in a flooded meadow near Villa Soriano, Uruguay (Photo: Martin Bollazzi). (C) Close up of another nest mound in the same region. The nest openings remain above the water level in the flooded meadow (Photo: Marin Bollazzi). (D) Another nest from Formosa, located beside a seasonal pond that is filled with water after spring rain.

The clayish soil, while dry and hard in the absence of rain, swells and becomes soft and plastic after wetting. It does not allow large quantities of rainwater to rapidly percolate, regularly resulting in temporal flooding of wide areas during the wet season. Flooded pastures and even deeper temporal ponds regularly appear in the immediate vicinity of *A. vollenweideri* nest mounts (Fig. 1). As a consequence, it can be assumed that *A. vollenweideri* colonies have to deal with strongly variable soil moisture conditions, which may significantly affect the growth rate of an established colony. In fact, long-term observations revealed that young nests grow primarily during the wet spring and summer season [15]. Occasionally, the upper layers are dry and hard, while deeper layers retain water. On other occasions, the top layer is extremely moist, or even covered by ponded water, while deeper layers remain dry, because water percolates through the E-horizon at a very low rate [11].

The idea that the water content of a soil has a strong effect on the growth rate of an ant colony is supported by the observation that leaf-cutting ants prefer high air humidities for fungus culturing [16]. While ants are expected to avoid dry soils because of the higher digging effort and risk of desiccation, soils with very high moisture appear to be unsuitable for nest building [17],[18]. In addition, workers of the red fire ant *Solenopsis invicta* Buren were even demonstrated to have an increased mortality at higher soil water content [19].

Intuitively, excess water can be disadvantageous as soon as it penetrates and accumulates inside nest cavities. The physical structure of the Gran Chaco clayish soils is known to prevent water percolation into the underground nest cavities, because the clay swelling creates an almost impermeable barrier for fluid flow. In fact, the nest chambers of *A. vollenweideri* are not coated with organic or inorganic linings [11], but they remain free of water even under a flooded nest surface. But surface water represents an important risk because of the water inflow via the nest openings. In such a case, low percolation rates would keep the inflowing water inside nest cavities and render them useless over extended periods. In mature colonies of *A. vollenweideri*, most of the nest openings are usually located above the local high water mark, particularly because they occur on top of the nest mound, or at least on the slopes [6]. Small colonies close all their nest openings during rainfalls, with the side effect that carbon dioxide concentration inside the nest increases significantly [8].

It is unknown when and how new tunnels and nest openings are constructed during nest growth. However, if nests mostly grow in the rainy season, there might be an imminent risk that new nest tunnels excavated upwards hit on accumulated surface water, with a concomitant water inflow into deeper nest chambers. Workers excavating new tunnels could hypothetically avoid the excavation underneath ponded water if their spatial memory and orientation abilities allowed them to know the locations of water ponds at the surface while digging underground. That seems unlikely, given the size and geometrical complexity of a mature *A. vollenweideri* nest. Alternatively, the ants could use the steep increase in soil moisture close to surface water as a cue to decide where to stop excavating. This leads to a situation in which a vertical moisture gradient would provide information influencing the workers' decisions as to where to excavate.

Considering the strong influence of water content on the physical properties of soil [20], ant excavation behaviour is expected to largely depend on soil moisture [21]–[23]. Sudd [21] described excavation behaviour in individual ants as a repetitive sequence of three consecutive behavioural elements, which have also been later described for *A. vollenweideri* in particular [24],[25]. (1) First, the soil is loosened at the digging face by ‘grabbing’ it with the mandibles. In *A. vollenweideri*, workers bite or cut into the material. (2) Then, the soil is aggregated into pellets by ‘raking’ by means of the mandibles and front legs. When *A. vollenweideri* workers rake moist clay, they rely mostly on pulling the material together with closed mandibles in the manner of an ice-cream scoop. During grabbing and raking the soil, and even slightly before, *A. vollenweideri* workers stridulate to attract nest mates to excavate at the same location [24]. (3) The resulting soil pellets are finally transported away from the excavation site. Soil transport becomes an increasingly time- and energy consuming task especially in larger nests with long distances in between the locations of underground digging and the nest openings. In *A. vollenweideri*, loads are transported sequentially, i.e. the excavator disposes of the carried soil pellet close to the digging site, where other individual picks a single pellet up for further transport [25].

Soil moisture may directly affect both the workers' digging performance and pellet transport rates. Moist soil may facilitate the steps of grabbing and raking [23] during the formation of a pellet. Additionally, moist soil particles tend to stick together and could be aggregated for transport [26],[27], probably resulting in much higher mass transport rates. On the other hand, moist soils may be easier to dig and carry, but because of their mechanical properties, the transmission of vibrational communication signals used to organize collective nest excavation [24] may be hindered due to higher attenuation rates.

Even though *A. vollenweideri* colonies are regularly exposed to varying moisture across the soil profile, it is an open question to what extent soil moisture influences workers' excavation performance, soil transportation and nest building. The present work was aimed at investigating these influences in a series of laboratory experiments using standardised clay mixtures with increasing water content, ranging from relatively hard to almost fluid material. We measured relevant physical properties (bulk density, toughness, tensile strength, pellet stickiness and attenuation rate for surface vibrations) of the used clay mixtures, and experimentally evaluated individual digging preferences, group-level working rates, vibrational recruitment behaviour and individual work performance. Finally, we investigated whether workers stop the excavation of an upward tunnel when approaching accumulated surface water, thus resulting in the prevention of water inflow into the nest.

## Methods

### Animals and Materials

Animals for the behavioural experiments were obtained from two different laboratory colonies of *A. vollenweideri*, excavated at an age of approximately 8 months at a privately owned site, the ‘Reserva El Bagual’, San Francisco de Laishi, Formosa, Argentina (26°17′08″ s; 58°49′43″ w), with the permission of Pablo Götz (owner) and Alejandro Di Giacomo (supervisor of the ‘Reserva’). *Atta vollenweideri* is widespread in the area and not protected under the Convention on International Trade in Endangered Species (CITES). No specific permits were required for the described studies. After excavation, colonies were transferred to the laboratory and kept under controlled conditions at 25°C and a 12∶12 h LD cycle.

While there is a considerable variation in the composition and physical properties of field soils, we concentrated only on the influence of varying moisture content. We therefore assayed 7 different standardised clay-water mixtures, based on industrial clay (CLAYTEC ‘Lehm gemahlen 10.001’) with a maximum particle size of 0.5 mm and varying only in water content. In the field, soils are sediment layers composed of variable proportions of clay, sand, silt and organic matter. Our choice may not necessarily match the characteristics of a particular field site [11], so that quantitative extrapolations to different soil types are not possible, i.e. a soil with 20% moisture may have very different physical properties at different locations. However, it allows the general qualitative effects of materials with relatively ‘high’ or ‘low’ moistures to be investigated under standardised conditions.

**Figure 2:**
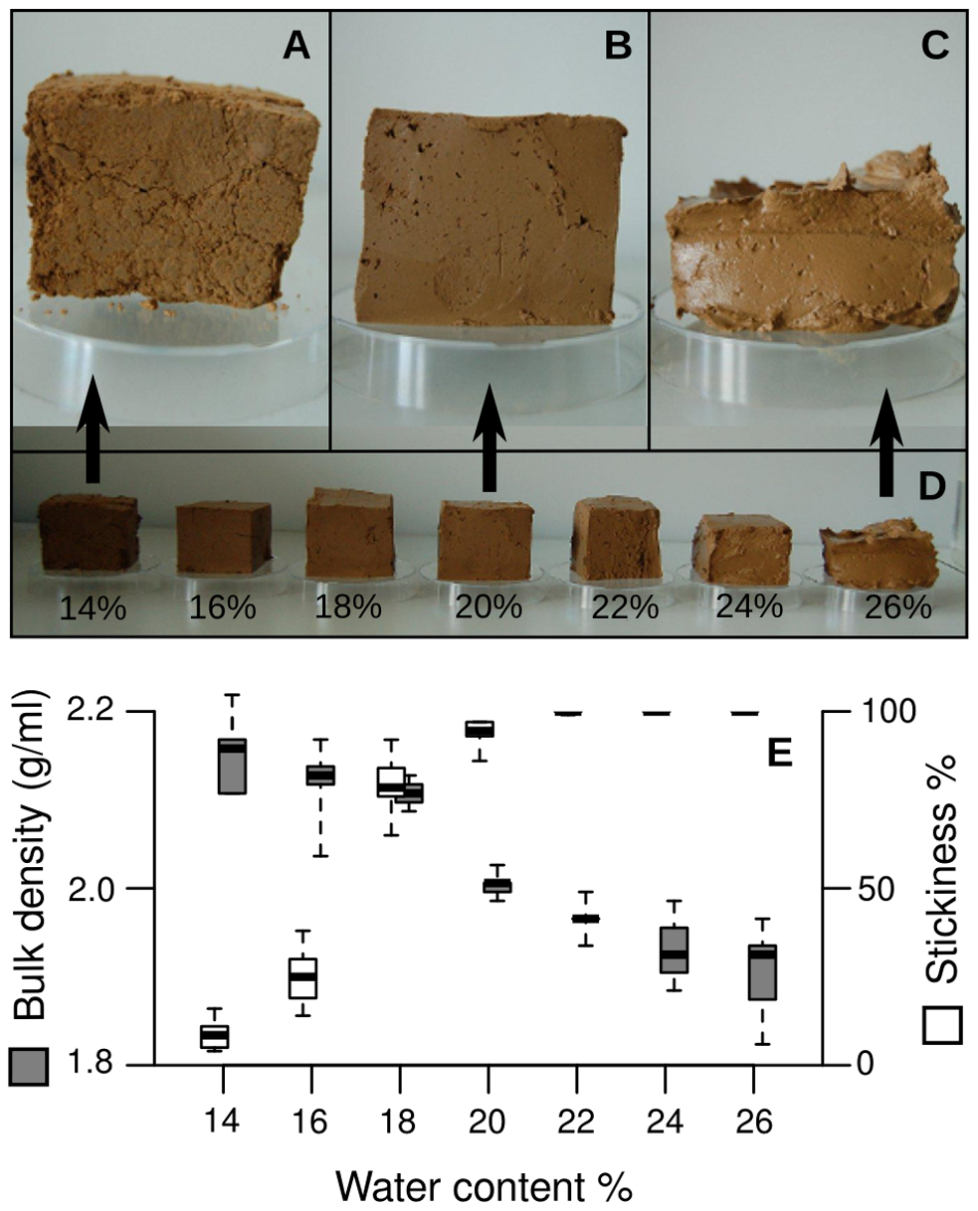
Clay mixtures used in the laboratory experiments. The water content in the seven mixtures ranged from 14% to 26% of the overall mass. (A–D) Samples of clay with 14% (A), 20% (B) and 26%(C) water content in close up, and the entire spectrum of mixtures used in this study (D), ranging from relatively hard and brittle to very soft and sticky material. The samples depicted here varied in mass. (E) Change of physical properties as a function of water content within the investigated range. Grey boxes represent the material bulk density of the material, scaled at the left Y-axis, white boxes represent the stickiness, i.e. the percentage of pellets from that mixture that stick to a surface of the same material. Stickiness values are scaled at the right Y-axis. Bars within the boxes indicate median values. The box shows the range from 25% to 75% quartile. Whiskers indicate minimum and maximum values without outliers. Sample size was *N = 10* for each measurement.

The gravimetric moisture contents (*u*) in each of the mixtures were 14%, 16%, 18%, 20%, 22%, 24% and 26% of the overall mass (Fig. 2A–D). Within this moisture spectrum, the physical properties of the material change considerably. However, it represents the maximum range of water contents that allows for the construction of the experimental set ups described below. At 14% water content, the material used in this study was dry and tough, but it could still be formed and pressed into vessels without breaking. With less water, the material becomes very brittle and impossible to mould homogeneously into the experimental set ups. At the maximum water content of 26%, the soil was sticky with a clearly developed water film at the surface, but still consistent enough to stay in place in an experimental set up. With higher water content, the soil resembled a thick fluid, making it impossible to create firm cavities. Accordingly, the moisture contents used in the experiment likely represent most of the range from the plastic limit to the liquid limit of the material.

Statistical analysis was done in R. 2.14.1 (http://www.r-project.org). For the sake of clarity, specific details of statistical methods used in the different experiments will be presented together with their results.

### Material properties

First, we quantified bulk density, toughness, ultimate tensile strength, pellet stickiness, and the attenuation rate of vibrational signals for each of the used clay mixtures.

Bulk density was determined by filling a 200 ml vessel with the material and by measuring the mass at nearest 0.1 mg. Density was calculated dividing the measured mass by 200 ml. Measurements were repeated five times for each of the seven mixtures.

To acquire an estimate of toughness, a plastic box of 9×9×6 cm was filled with the material to be measured. Then, an aluminium cuboid with a footprint of 1×1 cm was pushed into the soil from above with a constant force of 10 N. The penetration depth of the cuboid was measured at nearest 1 mm and used as a measure for soil toughness. Each measurement was repeated five times for each of the seven mixtures.

To measure ultimate tensile strength, two plastic tubes with an inner diameter of 22.1 mm were taped together and filled with the material. Then, the tubes were held vertically and the upper one was fixed on a retort (ring) stand. The tape was removed so that the lower of the two tubes was connected to the other one and held above the ground exclusively by the clay. A plastic box was attached to the bottom tube and slowly filled with sand. When the clay connection between the two tubes broke, the entire mass of the lower part of the construction, i.e. of the clay-filled tube plus the attached box and the amount of sand inside it, was measured at nearest 0.1 mg. This mass was considered the limit of the tensile force that can be held by the material. To calculate strength in N/cm^2^, this mass was multiplied by a gravitational force of 9.806 m/s^2^ and then divided by the cross section of 3.85 cm^2^.

The stickiness of the clay mixtures was assessed by determining if pellets of a particular moisture content adhered to the surface of the same material when held upside-down. For this experiment, a standard plastic Petri dish was filled with clay and the surface was smoothed. In separate box, ants were allowed to excavate the same material. Fresh pellets were taken and dropped onto the surface in the Petri dish from a height of about 1 cm. One hundred pellets were placed in that manner in one Petri dish. The Petri dish was then turned upside-down and held with the pellet-covered soil surface facing downwards. The proportion of pellets sticking to the soil surface without falling down was counted. This measurement was repeated 10 times for each of the seven moistures.

The attenuation rate of stridulatory signals involved in the collective organization of digging behaviour was determined for the different materials by measuring the remaining amplitude of an artificial wave of known original amplitude at different distances from the source. Attenuation in dB was then calculated at each distance, so as to determine a distance-dependent damping rate by regression analysis. The procedure has been described in detail in a former publication [24].

### Moisture preference

To investigate the preferred clay moisture to initiate digging in *A. vollenweideri* workers, their responses when presented with different soil moisture contents were quantified in two ways. In a choice experiment, all seven mixtures were presented simultaneously to determine the ants' preference to initiate digging. In a separate no-choice experiment, workers were presented with a single clay mixture and their stridulation rate while excavating was examined as a measure of recruiting intensity.

To investigate moisture preference, seven sample holders were prepared by cutting 1 cm long pieces from the top ends of photometric plastic vials. Each sample holder was filled completely with one of the clay mixtures. The clay faces were smoothed and the sample holders circularly arranged in a closed 5.5 cm diameter Petri dish. The dish had a hole in the centre of the bottom side that allowed the connection of flexible tubing with an inner diameter of 1 cm. The sample holders were arranged in a gapless circle with their clay faces oriented towards the centre hole and held in place with small pieces of play dough. Within that arrangement, the clay samples were sorted according to water content, the moisture increasing in clock-wise direction. The ants entered the experiment through a piece of tubing that connected an entire laboratory colony to the hole in the bottom of the Petri dish. Each replicate of the experiment was started by connecting the colony to the set up. The first occurrence of excavation behaviour at one of the samples was considered as a decision for this particular mixture.

This method, considering only the first response of the first worker within a group, allowed the observation of individual decisions without separating individuals from the colony and removing them from their social context. After a decision was observed, all parts of the set up were cleaned, the sample holders were refilled and the procedure repeated. Workers that had been within the set up during one replicate of the experiment were kept separately from the colony for the duration of the entire series. After each replicate, the position of the clay mixtures relative to the laboratory colony was rotated counter clockwise by 1/7 of the full circle, to rule out possible effects of the workers' walking direction when coming towards the set up on their preferred digging reaction. The whole procedure was repeated for *N* = 56 times.

Stridulation rates of excavating workers were measured for each clay mixture by carefully separating excavators in plastic vials, recording stridulation by means of an attached Bruel & Kjaer Type 4333 accelerometer, and counting the stridulation chirps produced per second while digging. The employed method has been described in detail in an earlier publication [24]. For each mixture, *N* = 10 workers were observed.

Differences in signal propagation that occur with varying moisture complicate measurement of stridulation rate. In materials with higher attenuation rates, stridulation signals are more likely to remain below the noise level and thereby be hidden from the observer. To compensate for the potential effects of different propagation properties, a reference measurement was conducted that replaced the ants in the experiment by a standardised playback signal. This artificial signal was applied to the clay surface in the experimental set up by means of an aluminium rod (55 mm long, 5 mm in diameter) connected to a Bruel & Kjaer Type 4810 mini shaker. The volume of the reference track was scaled so that the strongest signal in the track had a peak-to-peak amplitude of 8 cm/s^2^. The playback sample was 10 s long and contained 138 stridulation chirps. With each of the clay mixtures, the sample track was recorded again, and stored in a wave format audio file. The sound files were blinded for analysis, i.e. the files were randomly renamed by one person, while another person counted the signals. While no signal loss occurred at 14%, 16% and 18%, the number of counted signals decreased from the original 138 to 126 at 20%, 116 at 22%, 107 at 24% and 88 at 26% soil moisture content. The numbers were divided by the maximum of 138. This proportion provided the mixture-specific correction factor for signal counts.

### Group-level performance

In addition to the preference of workers that were able to choose, as described above, we investigated how group-level excavation rate is influenced by soil moisture if only a single mixture was available to the ants. Comparable experiments in several ant species, usually distinguishing only 2–3 levels of water content, reported faster excavation rates at higher moistures [21]–[23]. We temporarily separated subcolonies, i.e. groups of workers with a fungus garden, from the large laboratory colonies, and connected single subcolonies to a box entirely filled with a given clay mixture. The amount of soil removed from that clay box within 48 h was measured.

Subcolonies were obtained by detaching small plastic boxes of 9×9×6 cm that were completely filled with fungus garden from a large colony. Accordingly, both the number of workers and the amount of fungus in the subcolonies was only roughly standardised. We preferred that method over precisely determining the size of the subcolonies, because opening the fungus chambers to obtain material and separating ants from fungus for counting is so disturbing that the fungus may have reduced growth or even die if treated like that too often. To have a rough estimate of the number of workers in such a box, one was opened, the fungus taken apart and the number and size distribution of the workers inside investigated. As an example, it contained 912 workers, with a size distribution strongly biased towards small individuals. Even though this subcolony housed 397 workers larger than 0.9 mg, with 0.9 mg being the smallest worker size observed digging in later experiments, this number was considered as a sufficient work force for single digging experiments. The boxes were connected to the main colony for at least three days in advance before separation, then connected to the experimental clay box (18×9×6 cm) and an empty box for soil deposition (9×9×6 cm). The clay box was weighed before the experiment and 48 h after the subcolony had been connected. The experiment was repeated 51 times.

During the 48 h experiments, the clay in the boxes desiccated considerably. Because of variability among the replicates, even though the clay was initially prepared with defined moisture, it was impossible to assign each result to one of the initial well-defined mixtures, as for the other experiments. Therefore, moisture inside the boxes was measured after the experiments employing the ‘wet and dry’ method, i.e. taking a sample of about 400 g and determining its wet mass, its dry mass and comparing the two.

### Individual work performance

The effects of varying moisture and the associated changes in physical properties on individual work performance were investigated by comparing pellet size and working speed in both soil excavators and soil carriers. Excavators usually carried the pellets for few centimetres only. Other workers picked them up later on and covered the remaining distance to the nest opening. Accordingly, when looking at individuals, it is useful to distinguish soil excavation and soil transport as separate tasks [25]. We quantified digging and transport rates, tasks that require measuring the speed at which each item is processed (i.e. excavated or carried), and the mass of the item, using three different experimental set-ups.

In the first experiment, both pellet mass and pellet digging rate were quantified for excavating individuals. Seven plastic boxes of 9×9×6 cm were used, each one filled with one of the clay mixtures each. The clay in the boxes was 2 cm high only, leaving sufficient space for ant workers to move in the box on the clay surface where they still could be gently removed with tweezers for further measurements. A laboratory colony was connected to the box, whereupon workers entered it and started excavating. This construction allowed for the observation of individual excavators. The time they needed to excavate a soil pellet was measured, the animals and the produced soil pellets were collected and weighed. The number of observed workers at each moisture was *N* = 20, with the exception of 20%, where *N* = 40 workers were collected. Additional pellets were collected directly from their excavators for a more accurate measure of average pellet mass in between behavioural observations. As a result, about 70 pellets from each clay mixture were available for weighing.

The observation of soil transport speed was done in a different set up that allowed workers to excavate at the end of a clay tunnel and then walk through that tunnel carrying the excavated pellet. This tunnel was prepared in a clay layer between two horizontal glass plates of 20×10 cm. The tunnel started at the centre of one of the short sides of the construction, leading inwards parallel to the long sides for 15 cm. The tunnel had a quadratic cross section of 1×1 cm, and was located at the upper glass plate. The entire clay layer was 1.5 cm deep, thereby leaving 5 mm of soil between the ground of the tunnel and the bottom glass plate for the ants to walk on. Through the top glass plate ant workers were observed carrying soil pellets along the tunnel. This allowed the distance and duration that pellets were carried to be measured and the walking speed of the carriers to be calculated. The experiment allowed a large number of transport events to be observed (*N* = 83 at 14% and 18%, *N* = 84 at 24% and *N* = 100 at 16%, 20%, 22% and 26%), but did not allow the pellets to be collected afterwards for weighing.

Carried pellets were sampled, in yet another set-up, by filling entire plastic boxes with clay. These were connected to a second, open plastic box via 50 cm of tubing. The ants had to pass this second box on their way back from the clay box to the colony. Here, pellets were collected when carried out of the tubing and weighed. The rates at which pellets were carried out of the tubes differed considerably according to water content, resulting in different sample sizes between 11 and 24 for each clay mixture.

The results of these measurements showed no linear or exponential dependence on moisture. The water content was therefore treated as a factorial variable during the statistical analysis of these experiments, and the seven moisture grades from 14% to 26% as levels of that factor. As a result, the influence of moisture on the measured variables was tested with ANOVA, and differences in between the particular levels with Bonferroni-corrected pair-wise t-tests. Comparison of the masses of excavated and carried pellets was done by directly comparing the data sets level-wise with Welch two-sample t-tests. All data sets were *ln*-transformed in advance to compensate for light skewness.

### Prevention of water inflow

To investigate whether ant workers would continue to dig upwards irrespective of increasing soil moisture until meeting accumulated water or would stop before doing so, the situation of tunnelling upwards towards a seasonal accumulation of surface water was simulated in a laboratory experiment. Plastic tubes of 10 cm height and 4.5 cm inner diameter were filled 6 cm high with clay of 20% water content. A 1 cm clay layer with 26% moisture was added on top, and water was then carefully poured 2 cm high onto the soil. In a control series, no water was added on top of the soil. Before the experiment started, the filled tubes were stored for 24 h to establish a moisture gradient. The tubes were covered with plastic wrap for that time to minimise evaporation losses. For each replicate, 100 ant workers were placed in a plastic box of 9×9×6 cm. The box was connected to a T-shaped tube, one end pushed into the clay from below, allowing the ants to dig into the clay, the other end pointing downward. The bottom opening of the tube was closed with a fine metal wire netting, so that potentially inflowing water would not drown the ants. After 24 h, the ants and the water layer were removed from the set up. Beginning at the top, the soil was removed centimetre by centimetre. From each centimetre of clay, a sample was taken to determine its water content, and it was noted how far the workers had excavated upwards. In addition, the dry mass of the excavated soil pellets deposited in the plastic box was determined. Sample size was *N* = 17 for the experiments with water, *N* = 16 for the control without water.

## Results

### Material properties

Bulk density decreased from 2.2±0.1 g/ml (median ± interquartile range, *N* = 5) at 14% to 1.9±0.1 g/ml (*N* = 5) at 26% water content. This relationship was not linear. While decreasing constantly at a low rate from 14% to 18% and from 20% to 26%, bulk density was reduced considerably between 18% and 20% water content, indicating that the clay swells within that moisture range (Fig. 2E).

When measuring toughness, there was no measurable penetration of the material at moistures below 20%. At 20%, the aluminium cuboid penetrated the clay 1±0 mm (median±IQR, *N* = 5) deep. Penetration depth increased to 5±1 mm (*N* = 5) at 22% and finally to 23±3 mm (*N* = 5) at 24%. The 26% mixture was so soft that the aluminium cuboid, as well as the weight providing the force, sunk into the material completely until reaching the bottom of the clay box. The 26% clay must therefore be considered outside the scale of measurements.

Tensile strength decreased linearly (linear regression: *TS* = 0.97–0.02·*u*, *N* = 35, *R* ^2^ = 0.40, *P*<0.001) from 0.59±0.04 N/cm^2^ (median±IQR, *N* = 5) at 14% to 0.32±0.03 N/cm^2^ (*N* = 5) at 26% water content.

Regarding pellet stickiness, 8.5±0.5% (median±IQR, *N* = 10) of the pellets adhered to the surface even at lowest moisture of 14%. The proportion increased to reach 100% at 22% water content and remained at 100% for higher moistures (Fig. 2E).

The method for measuring the attenuation rates of substrate vibration was successful at moistures ranging from 14% to 20%. Within that range, the attenuation rate increased from 0.5 dB/cm at 14% water content, over 1.1 dB/cm at 16% and 18%, to 2.6 dB/cm at 20%.

The average signal peak-to-peak amplitude of a stridulation signal from a *A. vollenweideri* digging worker was 9.6 cm/s^2^, measured at a distance of 1 cm from the source. The reported maximum amplitude averages 21.7 cm/s^2^ [24], with a minimum physiological detection threshold for surface vibrations of about 4 cm/s^2^ in *Atta cephalotes* (L.) workers [28]. Accordingly, the propagation distance of an average signal decreased with increasing moisture from 8.6 cm at 14% water content to 2.5 cm at 20%. The potential range of the strongest signal decreased from 15.7 cm to 3.8 cm within the same moisture range.

At higher moistures, amplitudes remained below the noise level beyond a distance of one centimetre, so that the measurements were insufficient to calculate attenuation rates from regression analysis.

### Moisture preference, group performance and stridulation

**Figure 3:**
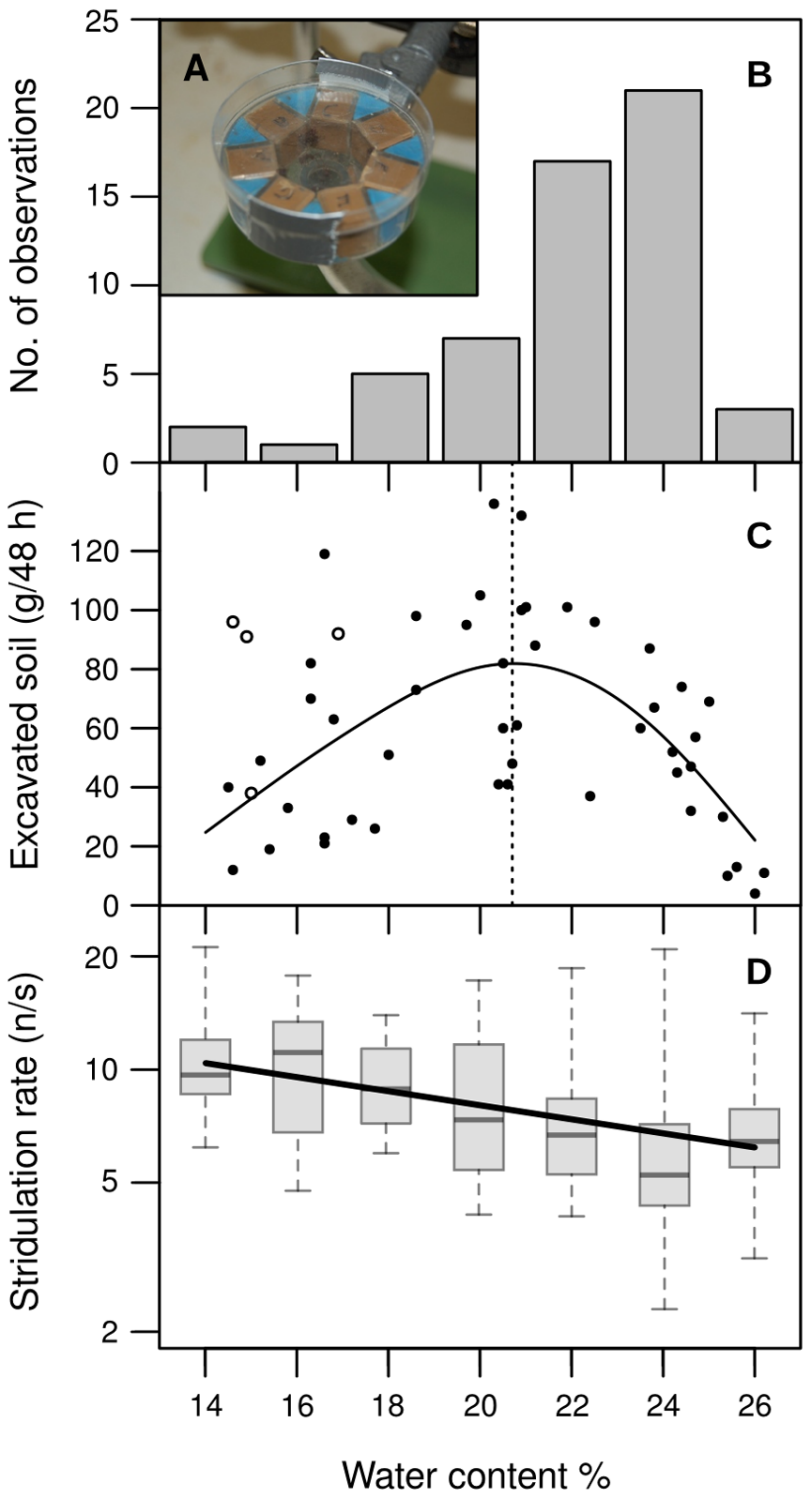
Individual preference and group-level performance. (A) Experimental setup for choice experiments on moisture preference. Seven clay samples of different water content were presented simultaneously to the colony. The first digging reaction on one of the freshly prepared samples was counted as a decision for that moisture. *N* = 56 decisions were observed. (B) Number of decisions for one of the presented clay samples as a function of moisture. (C) Group-level excavation effort as a function of soil moisture. White circles represent replicates in which workers relocated part of the colony fungus into the excavation chamber. These were not considered for statistical analysis. The black line shows the prediction of the generalized additive model fitted to the data (Deviance explained  = 41.1%, GVC score  = 772.13, Scale estimate  = 711.7, *N* = 47). The dotted line depicts the maximum predicted by the model. (D) Stridulation rate in signals per second (*r*) as a function of clay moisture (*u*). Stridulation rates were corrected for differences in signal transmission as outlined in the methods section. The Y-axis is depicted in logarithmic scale. Bars within the boxes indicate median values. The box shows the range from 25% to 75% quartile. Whiskers indicate minimum and maximum values without outliers. The solid line represents a linear regression of the *ln*-transformed stridulation rates (*r* = 19.1 · e^−0.04·*u*^, *R* ^2^ = 0.13, *P* = 0.002).

When presented with all seven mixtures simultaneously (Fig. 3A), workers first walked around in the narrow space in between the samples. It took between 1 and 5 minutes until the first digging response was observed. The ants mostly chose 24% clay moisture. This occurred 21 times, equalling 37.5% of the 56 experiments. 22% water content was chosen second most often, 17 times altogether (i.e. in 30% of the experiments). In general, preference, i.e. the number of decisions for a particular mixture, increased with increasing moisture (Fig. 3B), with the remarkable exception of 26% clay, that was chosen only 3 times (5% of the replicates). The observed distribution of choices deviated significantly from an even distribution (Chi-square test for goodness of fit: *Χ^2^* = 46.2, df = 6, *P*<0.001). In summary, preference depended on water content and increased with increasing moisture, but was very low at the highest water content of 26%.

Concerning the group-level excavation rate, results showed an increase of the cumulative excavated mass towards the middle of the tested moisture range, with a maximum of 136 g at 20.3% water content, and a minimum of only 4 g at the highest moisture of 26.0% (Fig. 3C). In four cases, fungus had been relocated into the clay box resulting in small cavities emerging around the fungus garden in the soil. As the direct presence of fungus can be expected to influence excavation rates [29],[30], these replicates were not further considered.

Identification of the moisture leading to the highest average group-level excavation rates was achieved by fitting a generalized additive model (gam) and computing the maximum of the predicted values and the corresponding moisture. Moisture was treated as a linear explanatory variable for the amount of excavated soil. According to the model, the excavated amount of soil was significantly influenced by water content (*N* = 47, *F*_2.7,3.3_ = 8.0, *P*<0.001). The fitted curve (the model explains 41.4% of the deviance) reached a maximum at 20.7% water content with a predicted value of 81.9 g (Fig. 3C).

Stridulation rates decreased with increasing moisture (linear regression, signal counts were *ln-*transformed to obtain normal distribution in the residuals: *F*_1,68_ = 10.5, *P* = 0.002). Average stridulation rate decreased from 9.7±3.1 signals/s (median±IQR, *N* = 10) at 14% moisture to 6.4±2.0 signals/s (*N* = 10) at 26% (Fig. 3D).

### Individual work performance

**Figure 4:**
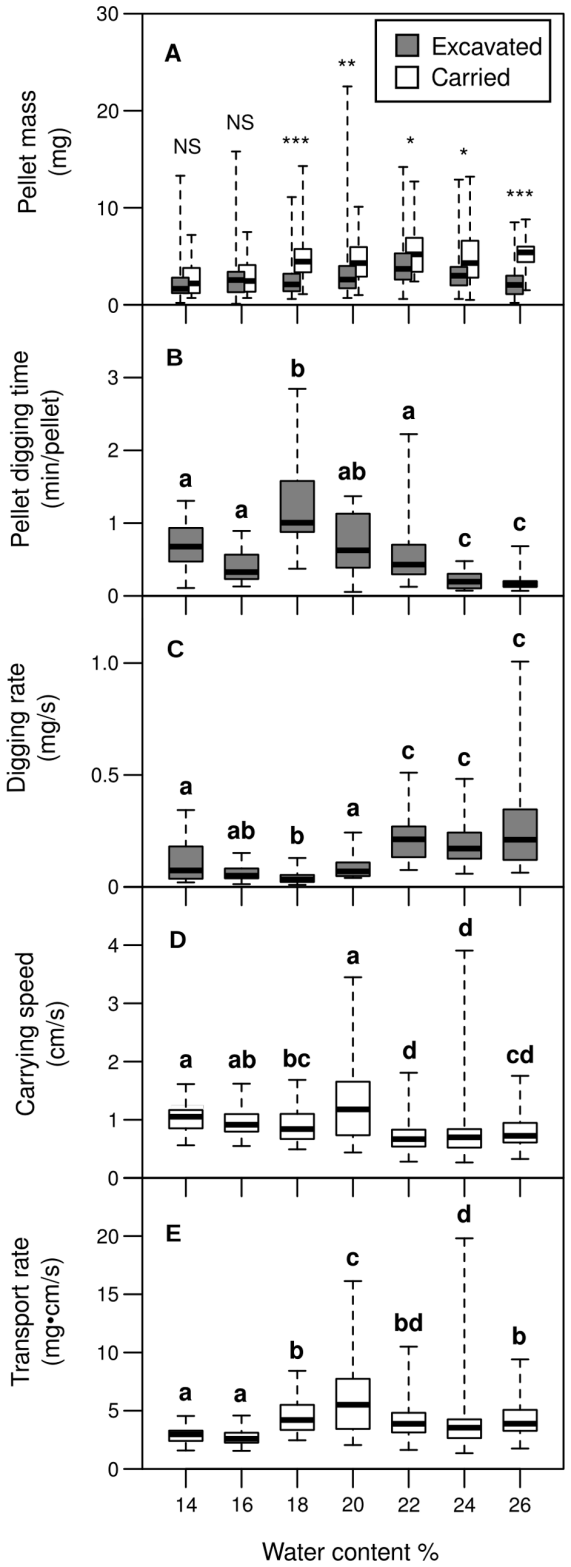
Excavation and transport performance as a function of water content. Grey boxes represent measurements associated with excavation, white boxes measurements associated with the transport process. Bars within the boxes indicate median values. The box shows the range from 25% to 75% quartile. Whiskers indicate minimum and maximum values without outliers. (A) Pellet mass of excavated and carried pellets for each clay mixture. Significance levels for differences between excavated and carried pellets are indicated above the Boxes (‘NS’ not significant, ‘*’ P<0.05, ‘**’ P<0.01, ‘***’ P<0.001, see Supporting Information, Tab. S1 for detailed statistics). (B) The time spent by a single worker to excavate a single pellet. (C) Digging rate calculated by dividing the mass of a freshly excavated pellet by the time spent by the worker to excavate it. (D) Walking speed of pellet carriers in a clay tunnel. In this case, the carried pellets were of the same mixture that composed the underground the workers had to walk on. (E) Transport rate calculated as the product of carrying speed and average pellet mass. Boxes labelled with the same letter are not significantly different. Details on the post-hoc comparisons are provided in Table S2–S5.

Excavated and carried pellets had a similar mass of 2.2±2.1 mg at 14% and 16% water content (median±IQR, *N* = 174). At moistures larger than 16%, carried pellets were significantly heavier when transported than when freshly excavated (Fig. 4A, for statistical details see Tab. S1), with a mass of 4.9±3.2 mg (*N* = 94) for carried, and 2.7±2.3 mg (*N* = 353) for excavated pellets. In both excavated and carried pellets, mass was significantly influenced by moisture (one-way ANOVA for freshly excavated pellets: *F*_6,486_ = 8.7, *P*<0.001; carried pellets: *F*_6,121_ = 5.3, *P*<0.001). The heaviest pellets were excavated and carried in mixtures with 22% moisture, with the excavated pellets averaging 3.7±2.7 mg (*N* = 74), the carried ones 5.2±3.1 mg (*N* = 20).

The time spent to excavate a pellet was also influenced by moisture (one-way ANOVA: *F*_6,137_ = 23.9, *P*<0.001). It took longest at 18% water content, in average 60.3±41.6 s (median±IQR, *N* = 20). In dryer soils, the time invested to form a pellet was only 40.7±24.4 s (*N* = 40), while at higher moistures, it decreased with increasing water content, to 9.3±4.1 s (*N* = 20) at 26% (Fig. 4B, see Tab. S2 for detailed statistics).

The individual excavation rate for each pellet, calculated by dividing pellet mass by the time spent to excavate it, also depended on moisture (one-way ANOVA: *F*_6,137_ = 23.9, *P*<0.001). The rate decreased from a median of 0.07±0.13 mg/s (median±IQR, *N* = 20) at 14% moisture to 0.03±0.03 mg/s (*N* = 20) at 18%. At higher moistures, the rate increased again, remaining relatively constant at 0.20±0.16 mg/s (*N* = 64) at a moisture equal or higher than 22% (Fig. 4C, see Tab. S3 for detailed statistics).

Median worker mass was 3.0±1.8 mg (median±IQR, *N* = 160). After *ln* transformation of body mass, a negative correlation of worker mass and moisture could be demonstrated by linear regression analysis (*F*_1,158_ = 85.3, *P* = 0.022), yet, though significant, the effect was weak (*m* = 4.39·*e* ^−0.02·*u*^, *R* ^2^ = 0.03). Median body mass decreased from 3.1±1.1 mg (*N* = 20) at 14%, to 2.3±0.9 mg (*N* = 20) at 26% water content.

Walking speed of pellet carriers was also influenced by moisture (one-way ANOVA: *F*_6,643_ = 33.5, *P*<0.001), the maximum being 1.2±0.9 cm/s at 20% (median±IQR, *N* = 100). On average, carriers moved at a slightly faster pace (0.9±0.4 cm/s, *N* = 266) at moistures below 20% than they did at moistures above 20% (0.7±0.3 cm/s, *N* = 284, Fig. 4D, see Tab. S4 for detailed statistics).

As it was not possible to weigh the pellets actually carried during the speed measurements, transport rates had to be calculated by multiplying the measured walking speeds with the moisture-dependent median pellets masses for carried pellets described above. The transport rates depended on water content too (one-way ANOVA: *F*_6,643_ = 49.1, *P*<0.001). As for the carrying speeds, the maximum was observed at 20%, in this case 6.0±3.0 mg·cm/s (median±IQR, *N* = 100). However, transport rates were generally lower in dry soil of 16% water content and less, averaging 2.7±0.9 mg·cm/s (*N* = 183), as opposed to 4.0±2.2 mg·cm/s (*N* = 467) at moistures of 18% or higher (Fig. 4E, see Tab. S5 for detailed statistics).

### Prevention of water inflow

**Figure 5:**
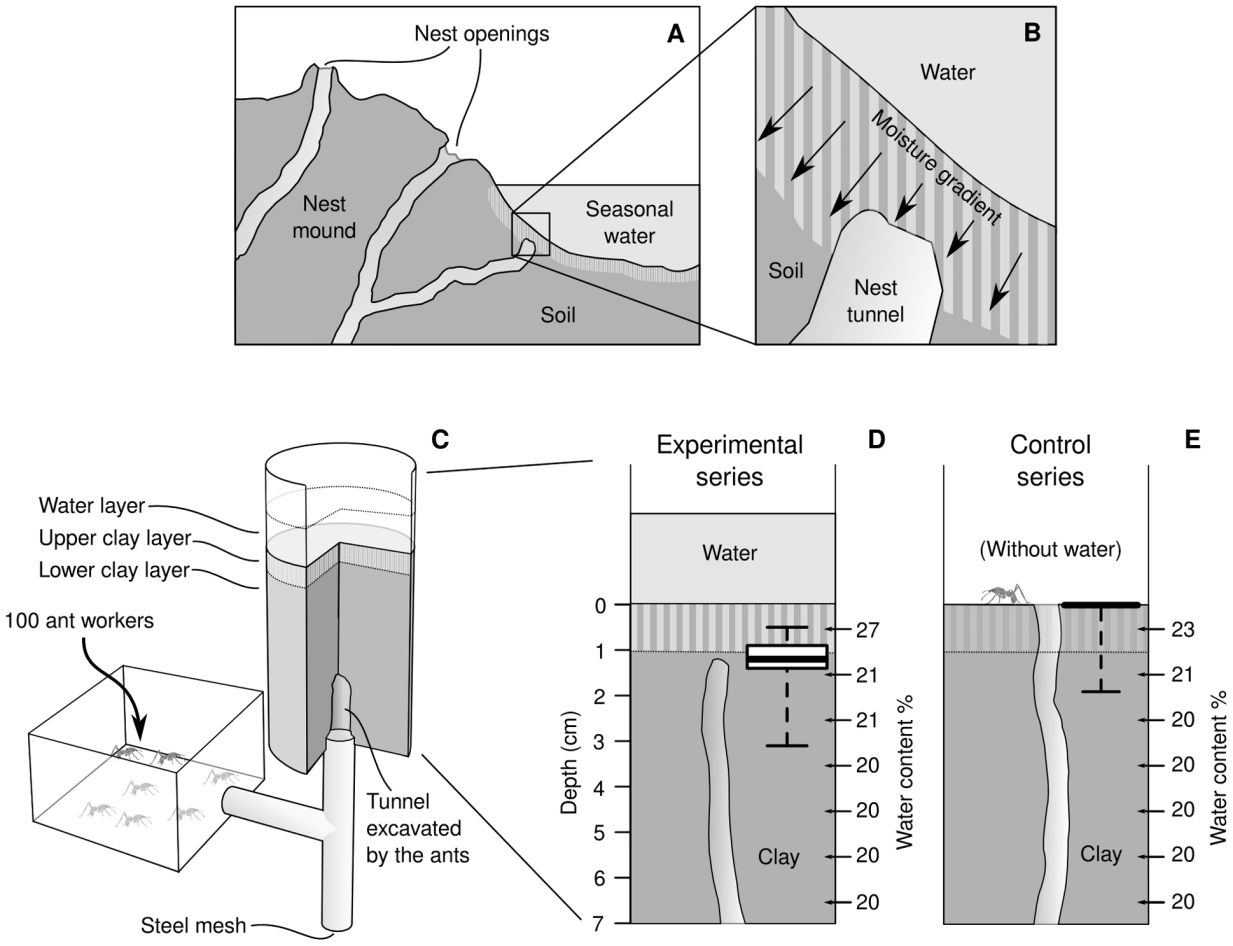
Avoidance water inflow. (A and B) Hypothetical situation in a field nest when water remains at the surface after a rain: a tunnel being excavated upwards to the surface reaches a layer of clay infiltrated by surface water. Further extension of the tunnel would result in water inflow into the deeper parts of the nest. (C) Experimental setup to simulate that situation in the laboratory. (D and E) Result of the experimental series and the control series without water: position of the tunnel end after 24 h. The left Y-axis represents the depth below the surface, the right Y-axis the median water content. Bars within the boxes indicate median values. The box shows the range from 25% to 75% quartile. Whiskers indicate minimum and maximum values without outliers. Sample size was *N* = 17 for the experiment and *N* = 16 for the control. Ant drawings were made by Karin Moll.

Simulating a situation with temporal accumulation of surface water above an underground nest structure in the laboratory (Fig. 5A–C), the observed excavated structures were exclusively vertical tunnels leading upwards through the clay. These tunnels reached the surface in all but two replicates (14 of 16) of the control series, while excavation was always stopped below the surface when water was present, at an average depth of 1.2±0.5 cm (median±IQR, *N* = 17; Fig. 5D–E), with the resulting tunnels being significantly shorter than the control ones (Wilcoxon rank sum test: *W*_17,16_ = 1127, *P*<0.001). However, the amount of soil excavated in the control series was not significantly higher (a mean of 15.1±11.7 g with water, 13.8±8.4 g without; Welch two-sample t-test: *t*_29.017_ = 0.4, *P* = 0.727), indicating that tunnels were not shorter just because the workers excavated at a lower rate under the conditions encountered when water was present at the surface. Water content was 26.5±1.0% (median±IQR, *N* = 17) in the uppermost soil layer when covered with water. One centimetre below, moisture strongly decreased to 20.5±0.3% (*N* = 17), evincing a very steep moisture gradient. In the controls, the moisture gradient started with 22.7±1.0% (*N* = 16) at the surface and decreased to 19.8±1.1% (*N* = 16) at 1 cm depth. To calculate the moisture value at which the ants stopped tunnel excavation in the experimental series, a four-parameter logistic model was fitted with the measured water contents (*u* in % of the wet mass) as the dependent variable, and average depth (*d* in cm below the surface) as linear predictor. The upper asymptote was assumed to equal a water content of 100% above the soil layer. As water content was measured as average values for a layer of 1 cm, the mean between the upper and the lower limit of the measured layer was used as a value for average depth, e.g. 0.5 cm for the upper layer from 0–1 cm, 1.5 cm for the next layer between 1 and 2 cm, and so on. According to the model, water content at 1.2 cm depth, where the tunnels ended in average, had an expected value of 21.1% (four-parameter logistic model, *u* = A+((B–A)/(1+e∧(-(D-*d*)/C))), with *A* = 100, *B* = 20.2, *C* = −0.38, *D* = 0.35; *F*_2,118_ = 274.4, *P*<0.001).

## Discussion

Underground-nesting ants are expected to excavate and build their nests at those soil layers providing a proper microclimate for colony growth, with the microclimate being determined by temperature, air composition, and moisture [8],[16],[19],[31]–[34]. In particular, the adaptation to specific soil properties has been suggested as a major explaining factor for the distribution pattern of leaf-cutting ant nests, an idea termed the ‘suitable soil’ hypothesis [35]. Regarding the relevance of soil moisture for nesting behaviour in *A. vollenweideri*, we have shown that the effects of soil moisture largely depend on the range under consideration, and that workers, as outlined below, do not necessarily prefer to dig at locations with the highest water content, which may represent an adaptation to avoid water inflow during seasonal floods.

### Transport rate and colony-level performance

Within the offered range of clay moistures, group-level excavation rate reached a maximum at 20% water content, while in the choice experiment, workers clearly preferred 22% and 24% moisture over 20%. This indicates that the maximum group-level excavation rate cannot simply be explained by individual preferences. Likewise, digging rate at the individual level was still comparably low at 20%, not reaching its plateau until a moisture of 22%. While neither preference, nor digging performance can account for the highest group-level excavations rates that occur at 20%, transport rate, intriguingly, had its maximum at the very same moisture. It is therefore argued that the rate at which ant groups remove soil from a box, a measure regularly used in laboratory experiments to asses ant excavation rates [22], [36]–[38], is limited mainly by the rate of soil transport rather than by the rate of loosening it from the surrounding walls. This view is supported by the observation that soil pellets accumulate inside nests when workers excavate [25],[29]–[30], indicating that soil pellets are usually produced at a rate higher than their rate of removal from the digging site.

Transport rates are probably higher at higher water contents due to soil pellets sticking together, thereby allowing the formation of larger loads. It seems as if that effect is especially strong in clayish soils. A comparable effect has also been described for desert ants inhabiting sandy soils. But here, workers use specialised “buckets” (e.g. psammophores) to keep their loads of sand grains together. Using these, they can aggregate moist sand grains for transport, which is not possible with dry sand [26],[27]. Here too, moisture seems to have a key role in load aggregation, but due to the lack of comparative data it remains unknown if that effect is stronger in clayish soils.

In our study, pellet stickiness strongly increases between 16% and 18% water content in the laboratory clay mixture. Comparing the masses of freshly excavated pellets and carried loads revealed the latter to be significantly larger, but only for moistures of 18% or larger, indicating that soil particles are carried in larger loads if they are sticky enough to be aggregated. In our study, that increase of load size had, for six of seven tested mixtures at least, a stronger effect on transport rates than the observed differences in carrier walking speed. The only exception was the clay with 20% water content, where large loads additionally combined with exceptionally fast carrying, resulted in the highest average transport rates observed during the experiments. It is possible that carriers moved at the fastest pace on the clay with 20% water content due to improved adhesion/detachment while walking, which would also be influenced by water content. However, it remains an open question.

### Soil moisture and vibrational communication

*Atta vollenweideri* workers stridulate while excavating and thereby attract nestmates to dig at the same location [24]. Our experiments have shown a negative influence of soil moisture on signal transmission. Maximum range decreased from about 15.7 cm to 3.8 cm with increasing moisture contents from 14% to 20%. In moister soils, attenuation was even too strong to be measured with the employed method. A positive influence of enhanced transmission of recruitment signals on group-level excavation rate was not observed. Considering the high group-level excavation rates at moistures of 20% and higher, it is concluded that hampered recruitment due to high attenuation rates at high moistures does not have a strong effect on overall group performance.

Moreover, the measured stridulation rates decreased slightly with increasing moisture, even after correction for signal loss due to moisture-dependent attenuation rates. Stridulation intensity thereby did not mirror the preferences of the workers. The weak decrease can possibly be explained by considering a poorer body-to-ground conduction on softer material: workers would apply more force when manipulating harder material, perhaps resulting in a better transmission of vibrations from the body to the material, while more signals are lost when the soil is softer. Such an effect cannot be completely compensated with the adjustments applied to the measured stridulations.

On the other hand, there are possible interpretations of increased stridulation rates in dry soils. Lower stridulation rates in moist soils can be interpreted as an adaptive response to poor transmission properties, if assuming that workers vary their stridulatory behaviour based on the actual soil properties, i.e. they stridulate less when the environment is less suitable for vibrational recruitment. Alternatively, high stridulation rates in dry soils can be seen as a reaction to materials that are harder to excavate, so that workers may increase their recruitment efforts to attract workers. Finally, the influence of soil moisture on digging stridulations can also be interpreted without considering any communicational aspect of stridulation behaviour. Spangler [39] hypothesized that stridulation in digging Hymenoptera was a mechanism that mechanically facilitated the loosening of the soil to be excavated. Following that hypothesis, the excavation of dryer, harder soils possibly requires more stridulatory vibrations. Digging stridulations have been demonstrated to serve a communicational function in *A. vollenweideri* [24], nonetheless, an additional mechanical function cannot be ruled out. However, regardless of all the efforts to compensate for moisture-dependent attenuation effect in the present study, the observed influence of moisture on stridulation rates can still be a direct result of different propagation properties.

### Inflow prevention

Workers excavating an upward tunnel below accumulated surface water were observed to stop digging before reaching the interface soil/water. Why do workers stop digging before reaching the accumulated water? The results of the previous experiments indicated that both excavation and transport rate are consistently high at moistures higher than 20% water content. The choice of the individuals, if presented with different moistures, seemed to be dominated by the preference for materials that can be excavated at higher rates. In the choice experiments, most workers selected clay with 22% and 24% water content. Both values fall into the moisture range that allows the highest excavation rate, indicating that preferring such a soil over a dryer alternative for digging can save time and, due to the decreased toughness and tensile strength, possibly even reduce energy investments. But, although clays with 26% water content were excavated and transported at the same rate, workers rarely preferred that mixture. Additionally, the lowest group-level excavation rates were observed close to 26% water content, most probably as a result of avoidance by the workers, according to the above considerations.

Avoidance of the highest moisture presented cannot be simply explained by physical properties and their influence on work performance. It can be, nevertheless, possibly explained by considering high moistures as an indicator of proximity to water accumulations such as seasonal ponds, so that further excavation could in some cases provoke water inflow into the nest. Our experiment on the prevention of water inflow, which confronted workers with a very steep moisture gradient, demonstrates the ability of *A. vollenweideri* workers to stop tunnelling upwards before reaching ponded water at the surface. Interestingly, although the 26% clay mixture was not completely rejected during the choice experiments, workers stopped digging at much lower moistures in all of the gradient experiments, around 21.1% based on our calculations, so that water inflow was successfully prevented. The average water content in the clay mixture decreased from 27% in the first centimetre to 21% in the second. It is therefore suggested that workers responded to the moisture gradient rather than to a specific threshold moisture to stop digging, and that they were able to distinguish moisture changes at a relatively small spatial scale. The comparison of the observed value, i.e. 21.1%, with the results of the choice experiment, where clays of 22% and 24% were highly preferred, suggests that workers indeed respond to the gradient rather than to a particular threshold value when deciding to stop excavation.

Admittedly, our measurements of the moisture gradient, yielding centimetre-wise averages, were of limited precision, and gave a poor representation of the moisture changes over distance. Nevertheless, the measurements show that the standing water does not percolate very deep into the clay, reinforcing the idea that ants need to respond to small-scale moisture gradients.

The situation discussed here, a tunnel excavated upwards in the direction of standing water that seasonally accumulates at the surface, is certainly a hypothetical one. Nonetheless it is plausible considering field observations, and the results of the gradient experiment clearly demonstrate the ants' ability to avoid inflow of surface water when digging towards accumulated water. It remains an open question how new nest openings develop during colony growth, but the experiment demonstrates that tunnels can be built from the underground to the surface even in the rainy season without the risk of excavating upwards underneath water that may inflow into the nest. It is important to emphasize that clayish soils, on the one side, may prevent surface water to enter the nest, but on the other side preclude a rapid water percolation in the case of partial nest flooding, rendering the flooded areas of the nest useless for prolonged periods. Because of the observation of water-free nest cavities immediately adjacent to ponded water in field *A. vollenweideri* nests, Jonkman [6] supposed that the inner walls of nest chambers were waterproof, coated with organic matter as reported for termites. However, micromorphological analyses showed no lining of the chamber walls at all [11]. The capillary forces in the clay-heavy soil of the Gran Chaco are probably sufficient to prevent extensive water movement through soil layers. Other phenomena have been reported that can be interpreted as mechanisms to prevent water inflow in *A. vollenweideri* nests. Young colonies with shallow nest mounds were observed to close their nest openings completely during rainfalls [8]. Older nests have higher nest mounds and the openings are usually located above the ground level. Within the nest mounds, blindly ending tunnels indicate former nest openings that have been closed completely and never used again during the ontogeny of the nest [6],[15].

### Water and nest ontogeny

Moisture gradients across the soil profile have been suggested to influence the digging activity of ants, and therefore the final nest shape. Desert-dwelling harvesting ants, *Messor ebenius* Forel, appear to remove wet sand when water has percolated into the nest, but stop as soon as only dry soil is left around them [40]. Accordingly, nest shape in this species may be mostly determined by the infiltration behaviour of the water. Our observation that *A. vollenweideri* workers prefer to excavate in moist soils that allow for high digging and transport rates, but avoid excavation beyond a particular point along an increasing moisture gradient, can therefore provide a framework for the understanding of some morphological features previously reported for *Atta* nests. Two old publications documenting systematic excavations of *Atta* nests described vertical tunnels leading into soil layers below the level of the fungus chambers [41],[42]. Other studies even described very large cavities, roughly bell-shaped [4]–[6],[43], generally spatially separated from the main location of fungus chambers, sometimes located at deeper soil layers, and often morphologically different from the latter. Such vertical, large cavity-like structures were not found in a number of other studies on the same and on other *Atta* species [1],[2],[44]–[46], and it remains an open question whether they occur only depending on particular local soil conditions. It has been hypothesized [4]–[5] that such deeper structures, with or without cavities, reach down to the groundwater table, or at least to the depth the groundwater level had at the time of their construction. Deep cavities in *Atta sexdens* and *Atta cephalotes* nests were often filled with water or at least reached into layers of moist soil. Some of them were reported to be filled with the colony's refuse, and accordingly referred to as refuse chambers [4],[5], as for the same type of cavity in *Atta vollenweideri* [6]. Stahel & Geijskes [4],[5] distinguished the empty cavities from the refuse chambers, calling the empty ones ‘Zisternen’ (cisterns), and argued that they represented water reservoirs for colony needs. They suggested that by having access to an underground water source, the colony would be able to regulate air humidity in the fungus gardens by carrying either water or wet soil up into the fungus chambers.

In the light of these descriptions, it can be assumed that under some circumstances, the extension of vertical tunnels in both directions, upwards and downwards, until reaching either the surface or a soil layer with drastically increasing water content, is a general pattern in the construction of *Atta* nests. A moisture gradient in the soil can be expected to occur close to the groundwater table as well. While stopping an upwards excavation after contacting very moist soil can prevent water inflow into the nest, as demonstrated in our experiment, a similar response during a downward excavation can account for the supposed pattern of refuse chambers or cisterns reaching down to the groundwater level. The described bell-shape of the cavities may be a result of the ants excavating downwards in the moisture gradient. As in our experiments, they are likely to excavate more at higher moistures but to avoid the layers surpassing a particular threshold, thus excavating further centrifugally from the end of the tunnel, following their moisture preferences. Their excavation rates are expected to be high at higher moistures, i.e. at deeper soil layers close to the groundwater level, and lower at higher, dryer soil layers, thus resulting in the bell shape described in the publication. This mechanism provides, at least in some cases, a hypothetical explanation for the occurrence of giant chambers in *Atta* nests, which may remain empty or be used for the deposition of refuse. In that case, one environmental variable influencing the shape of the *Atta* nests would be the distribution of water in the soil, as has been suggested for the nests of *M. ebenius* [40]. Nevertheless, even though such responses to groundwater provide a persuasive interpretation of some aspects of *Atta* nest architecture reported in literature, the authors of the excavations poorly described how they actually measured the depth of the groundwater table, or whether they simply assumed its existence from their observations of the nest architecture. Thus, such interpretations remain purely hypothetical, and further investigations on the structure of field nests and soil features are required to fully understand the effect, if any, soil moisture has on the morphology of the deeper parts of the nest.

*Atta vollenweideri* nests were reported to grow mostly during the rainy season [15]. Beside seasonal variation in the growth rate of the colony because of increased harvesting, our results suggest that increased growth may also result from workers digging faster at higher water content. Additionally, our results demonstrate that a moisture gradient provides a cue that prompts workers not to excavate further when tunnelling towards surface water. Accordingly, it can be argued that soil moisture influences nest growth in *A. vollenweideri* in two ways: its annual variation is probably an important factor for nest growth in general by influencing excavation rates, and strong increases in moisture provide a local cue for excavating workers so as to stop tunnelling and prevent accidentally flooding the nest, the latter aspect likely being an important adaptation to the specific habitat of the Chaco leaf-cutting ant.

## Supporting Information

Table S1 Mass of excavated and carried pellets at different moistures.
(DOC)

Table S2 Digging duration as a function of moisture: post-hoc comparisons.
(DOC)

Table S3 Excavation rate as a function of moisture: post-hoc comparisons.
(DOC)

Table S4 Carrying speed as a function of moisture: post-hoc comparisons.
(DOC)

Table S5 Transport rate as a function of moisture: post-hoc comparisons.
(DOC)
