# Supplementary material for: Soil Moisture and Excavation Behaviour in the Chaco Leaf-Cutting Ant (Atta vollenweideri): Digging Performance and Prevention of Water Inflow into the Nest
Source: PLoS One. 2014 Apr 18;9(4):e95658. doi: 10.1371/journal.pone.0095658 (PMC3991694; doi:10.1371/journal.pone.0095658)
Supplement: Table S1 — Mass of excavated and carried pellets at different moistures. (DOC) [file pone.0095658.s001.doc]

**Table S1. Mass of excavated and carried pellets at different moistures.**

| Soil water content | Mean pellet mass (mg)¹ | | *t* | *df* | *P* |
| --- | --- | --- | --- | --- | --- |
|  | excavated | carried |  |  |  |
| 14% | 2.8±2.8 (70) | 2.8±2.0 (14) | 0.6 | 20.8 | 0.536 |
| 16% | 3.0±2.8 (70) | 2.8±1.8 (20) | 0.6 | 41.5 | 0.572 |
| 18% | 2.8±2.4 (69) | 5.0±3.0 (24) | 4.6 | 46.3 | <0.001 |
| 20% | 3.3±3.2 (70) | 4.7±2.5 (19) | 2.9 | 33.0 | 0.006 |
| 22% | 4.6±3.0 (74) | 5.8±2.9 (20) | 2.3 | 36.0 | 0.027 |
| 24% | 3.5±2.5 (70) | 5.1±3.0 (20) | 2.2 | 28.7 | 0.036 |
| 26% | 2.4±1.7 (70) | 5.4±2.0 (11) | 5.9 | 21.0 | <0.001 |

Excavated and carried pellets were compared at each moisture by means of Welch two sample t-tests, data sets were *ln*-transformed to approximate normal distribution. ¹ Mean±standard deviation, sample sizes are indicated in parentheses behind the values.
