# Supplementary material for: Soil Moisture and Excavation Behaviour in the Chaco Leaf-Cutting Ant (Atta vollenweideri): Digging Performance and Prevention of Water Inflow into the Nest
Source: PLoS One. 2014 Apr 18;9(4):e95658. doi: 10.1371/journal.pone.0095658 (PMC3991694; doi:10.1371/journal.pone.0095658)
Supplement: Table S2 — Digging duration as a function of moisture: post-hoc comparisons. (DOC) [file pone.0095658.s002.doc]

**Table S2. Digging duration as a function of moisture: post-hoc comparisons.**

Measurements were grouped according to soil water content and *ln*-transformed. Given are the adjusted *P* values of post-hoc pair-wise Welch two-sample tests.
